# The guarded engagement loop: risk salience and interaction-driven underperformance in generative AI adoption

**DOI:** 10.3389/frma.2026.1830196

**Published:** 2026-06-19

**Authors:** Connie Mosher Syharat, Arash Zaghi, Sarira Motaref

**Affiliations:** 1 College of Engineering, University of Connecticut, Storrs, CT, United States; 2 School of Civil and Environmental Engineering, University of Connecticut, Storrs, CT, United States

**Keywords:** algorithm aversion, generative AI adoption, guarded engagement loop, human-AI interaction, large language models, risk salience, trust in automation

## Abstract

Generative AI adoption is often framed primarily as a question of learning technical skills. It is thought that if users learn better prompting and evaluation practices, useful outputs will follow, leading to greater reliance on the technology. This perspective overlooks a defining feature of large language models (LLMs): their output quality depends heavily on how users engage with them. Because LLM performance varies substantially with depth of disclosure, contextual richness, and iterative refinement, user interaction strategies directly shape perceived usefulness and observed performance. This paper develops a conceptual framework that proposes how risk salience may shape these interaction dynamics. Drawing on research in trust in automation, privacy calculus, algorithm aversion, and the social amplification of risk, we propose the guarded engagement loop, a multilevel feedback mechanism in which risk perceptions may shape interaction strategies that influence observed performance and, in turn, recalibrate trust in generative AI systems. At the micro level, elevated risk salience related to privacy, safety, or ethical concerns may lead users to adopt guarded interaction strategies characterized by reduced contextual disclosure and limited iteration. These constrained interactions can lower output quality and increase the likelihood of visible errors, which may further erode trust and reinforce cautious engagement. At the macro level, values-driven withdrawal from AI use has the potential to narrow the diversity of visible applications, amplifying risk-focused narratives, reinforcing perceptions of harm in public discourse. The guarded engagement loop framework conceptualizes generative AI adoption as a feedback process in which risk perceptions may shape interaction conditions that, in turn, can influence observed performance and subsequent trust calibration. We articulate testable propositions and discuss implications for organizational governance, AI system design, and institutional conditions that enable bounded openness and calibrated reliance.

## 1 Introduction: the underperformance dynamic

Generative artificial intelligence (AI) adoption is often framed as a question of technical skill, with performance improvements attributed to better prompt construction and evaluation practices (Zamfirescu-Pereira et al., 2023). This perspective overlooks a core property of large language models (LLMs): output quality depends heavily on how users engage with them (Ma et al., 2025). Prompts structure the information a model receives and even small changes in instruction, context, or examples can substantially alter performance (Brown et al., 2020; Liu et al., 2023; Min et al., 2022; Wang et al., 2024). As a result, system performance is not fixed but varies with the conditions of interaction.

The central contribution of this paper is to theorize a proposed self-confirming performance-evidence mechanism in generative AI adoption. Users who perceive AI use as risky may evaluate the system under interaction conditions that they themselves, rationally and protectively, have constrained. A user concerned about privacy, stigma, accountability, or ethical legitimacy may withhold context, avoid exploration, or stop after a single weak output. The resulting output may then be interpreted as evidence of system unreliability rather than as evidence generated under low-context, low-iteration conditions. In this sense, interaction behavior does not merely accompany adoption; it shapes the performance evidence that users later use to calibrate trust. This dependence on user interaction behaviors may thus create a self-fulfilling dynamic. These dynamics are particularly consequential in research and scholarly contexts, where generative AI is increasingly used in writing, analysis, peer review, and research evaluation. In such settings, interaction conditions may shape not only individual outcomes but also the performance evidence used to assess the role of AI in research workflows.

Research in adjacent domains supports components of this dynamic. Work on privacy calculus and information disclosure suggests that perceived privacy risk reduces users' willingness to share contextual information (Dinev and Hart, 2006), which in the case of LLMs may further limit system effectiveness. Studies of algorithm aversion demonstrate that users often reduce reliance on algorithmic systems after observing errors, even when those systems outperform human alternatives (Dietvorst et al., 2015). These strands of research suggest how constrained interaction and error interpretation may contribute to declining trust over time (Lankton et al., 2016). In this way, public discourse defining the technology as risky becomes part of the interaction itself, shaping behavior in ways that influence subsequent outcomes (Merton, 1948).

These dynamics suggest that underperformance may emerge not only from skill gaps, but from feedback between perceived risk, interaction strategy, and evaluation. As a result, training alone may be insufficient to address adoption barriers. Adoption is shaped in part by safety and privacy concerns (Featherman and Pavlou, 2003), whose salience and perceived severity are amplified through institutional and media processes as described in Social Amplification of Risk Theory (Kasperson et al., 1988). This broader risk environment influences not only whether users engage with AI, but the conditions under which they do so. Existing work on AI adoption provides insight into how users evaluate system performance, but it often treats performance as a property of the system rather than as something that arises through interaction. The guarded engagement loop brings these dynamics together by linking risk salience, interaction strategy, performance outcomes, and trust calibration within a reinforcing feedback process.

This paper proposes a dynamic multilevel model of underperformance in probabilistic AI systems. We begin by examining how interaction-dependent performance challenges assumptions embedded in traditional technology adoption models. Building from this critique, we articulate a framework in which media framing and legitimacy dynamics shape perceived risk, organizational safeguards and psychological safety influence engagement conditions, and interaction strategy co-produces system performance at the user level. We then develop the guarded engagement loop to explain how users make sense of early AI performance, how trust is calibrated and recalibrated over time, and how these dynamics shape subsequent reliance or disuse. We conclude by discussing implications for AI training, organizational adoption, and the governance of probabilistic systems.

## 2 Limits of classic adoption models

### 2.1 Core constructs and conceptual definitions

The guarded engagement loop draws on several strands of research, including trust in automation, privacy calculus, and human-AI interaction. This section defines the core constructs used throughout the framework and clarifies their roles within the proposed model. These constructs operate at different points in the interaction process, including how risk is perceived, how users engage with the system, how interactions take shape, and how outcomes are evaluated and influence future engagement. Although these constructs are closely related, they operate at different points in the interaction process and are not interchangeable. The constructs are presented in the order in which they operate within the interaction process, from risk perception to engagement, interaction, and evaluation.

**Risk salience** refers to the degree to which potential negative consequences of AI use are cognitively prominent and perceived as relevant to the user (Featherman and Pavlou, 2003; Kasperson et al., 1988). The perceived importance of AI-associated risks may be informed by direct experience, institutional context, media framing, or public discourse. In this model, risk salience shapes how users approach interaction with the system.

**Guarded engagement** is defined as a pattern of interaction in which users limit how they engage with a generative AI system in response to perceived risk. In this framework, guarded engagement is treated as a multidimensional behavioral orientation rather than a single discrete behavior. It can be expressed through at least three distinct but related forms of constrained interaction: reduced contextual disclosure, in which users limit the amount or specificity of contextual information they provide; limited iteration, in which users reduce back-and-forth refinement; and avoidance of exploration, in which users avoid open-ended, creative, unfamiliar, or high-stakes uses. Rather than representing separate constructs, these dimensions are understood as interrelated manifestations of a broader orientation toward constrained interaction under perceived risk.

**Interaction richness** refers to the degree of informational and iterative depth present in a user-AI exchange. This concept draws on media richness theory from organizational research, which emphasizes the role of contextual detail and feedback in reducing ambiguity (Daft and Lengel, 1986), but is adapted here to reflect the distinctive properties of prompt-based interaction with generative AI systems. In this framework, interaction richness is shaped by two closely related aspects of the interaction: the extent and quality of contextual information provided, and the degree of iterative refinement over the course of the exchange. The first reflects how fully the user specifies goals, constraints, and relevant context, while the second reflects how much the user engages in back-and-forth development, clarification, and revision of outputs. In this context, interaction richness reflects the extent to which users provide contextual detail, articulate goals and constraints, and engage in iterative refinement (Liu et al., 2023; Zamfirescu-Pereira et al., 2023). Here, interaction richness is treated as a property of the interaction itself rather than as a behavior. Guarded engagement may reduce interaction richness by limiting one or both of these aspects, resulting in interactions that are either under-specified, minimally refined, or both. These aspects may vary independently in practice, allowing for different interaction profiles (e.g., high disclosure with low iteration, or vice versa). In this framework, it emerges from how users engage with the system, including the extent to which engagement is guarded or open.

**Performance outcomes** are considered in two distinct forms. First, **objective performance** refers to the actual quality or accuracy of AI output relative to task requirements or external criteria. Beyond this, **perceived performance** refers to the user's subjective evaluation of the usefulness, adequacy, or reliability of that output. It is important to note that objective and perceived performance may diverge, as users can underestimate or overestimate output quality depending on expectations, prior beliefs, and the richness of the interaction through which the output was produced (Lankton et al., 2016; Lee and See, 2004).

**Trust calibration** refers to the alignment between a user's level of trust in the AI system and the system's actual capabilities and limitations. In this framework, trust calibration is treated as a judgment that is updated based on perceived performance rather than as a direct reflection of objective performance. Miscalibration may result in under-reliance (disuse) or over-reliance (misuse), both of which are well documented in trust-in-automation research (Lee and See, 2004).

These constructs define the core mechanism of the proposed guarded engagement loop. Risk salience is expected to shape the likelihood that users adopt a guarded engagement strategy. Guarded engagement, in turn, may constrain interaction richness by limiting the amount of contextual information users provide and/or the extent to which they engage in iterative refinement. Reduced interaction richness is expected to influence objective performance, which users then interpret as perceived performance. These perceptions are likely to inform trust calibration, which in turn may shape future interaction strategies and engagement patterns. Because interaction richness depends on both the quality of information provided and the depth of iteration, different forms of guarded engagement may produce different interaction conditions and performance outcomes. These relationships describe a process in which interaction behavior shapes the conditions under which performance is produced and evaluated. Table 1 summarizes the key constructs, their definitions, and their roles within the framework.

**Table 1:** Core constructs in the guarded engagement loop and their roles within the proposed framework.

Construct	Definition	Role in model
Risk salience	The degree to which potential negative consequences of AI use are cognitively prominent and perceived as relevant to the user	Shapes how users approach interaction with the system
Guarded engagement	A pattern of interaction in which users limit how they engage with a generative AI system in response to perceived risk, expressed through reduced disclosure, limited iteration, and avoidance of exploration	Constrains how users interact with the system
Interaction richness	The informational and iterative depth of a user–AI exchange, reflecting the quality of contextual information provided and the degree of iterative refinement	Mediates how interaction affects performance
Objective performance	The quality or accuracy of AI output relative to task requirements or external criteria	Reflects system performance under given interaction conditions
Perceived performance	The user's subjective evaluation of the usefulness, adequacy, or reliability of the output	Drives evaluation and trust calibration
Trust calibration	The alignment between a user's level of trust and the system's actual capabilities and limitations	Shapes future interaction and engagement patterns

### 2.2 Acceptance models and probabilistic AI

Classic adoption theories such as the Technology Acceptance Model (TAM) emphasize perceived usefulness and ease of use as direct predictors of intention and use (Davis et al., 1989). The Unified Theory of Acceptance and Use of Technology (UTAUT) builds on this model, emphasizing performance expectancy, effort expectancy, social influence, and facilitating conditions (Venkatesh et al., 2003). These models emerged in contexts where many workplace technologies exhibited relatively stable behavior once learned, allowing users to determine capability from early experiences.

Generative AI challenges the assumption of stability in two ways. First, at the system level, performance is inherently probabilistic and context sensitive. Identical prompts can yield variation, and small changes in phrasing or contextual detail can produce markedly different outputs (Wei et al., 2022). Second, at the user level, performance depends on the development of interaction skills. Adoption is not merely a matter of initial exposure, but of learning how to structure prompts, provide relevant context, and iteratively refine outputs. As a result, early experiences may reflect both stochastic variability and user inexperience, making perceived usefulness particularly fragile in the initial stages of adoption. In probabilistic systems, early experiences can shape adoption trajectories: low-quality interactions early on can lower perceived usefulness even when the system is highly capable. Reliance on such systems may then become less a matter of initial acceptance and more a question of gradual calibration of trust through repeated interaction (Lee and See, 2004).

### 2.3 Interaction richness and perceived usefulness

In the case of LLMs, perceived usefulness depends not only on the system's underlying capability but also on the richness of the interaction. Prior research suggests that interaction richness is shaped by the extent to which users provide goals, constraints, examples, domain context, and iterative feedback. Media Richness Theory proposes that richer communication channels in organizations better resolve ambiguity by enabling feedback and nuance (Daft and Lengel, 1986). Similarly, LLM interactions can support “rich” exchanges, but only when users supply sufficient context and engage iteratively rather than expecting definitive answers from a single prompt.

Empirical work in adjacent domains provides evidence relevant to this dynamic. Research on the personalization-privacy paradox shows that when users perceive privacy risks, they disclose less contextual information, which in turn reduces system effectiveness (Xu et al., 2011). Similarly, studies of LLM use demonstrate that output quality is highly sensitive to prompt structure and that non-expert users often struggle to construct prompts that elicit the system's full capabilities (Zamfirescu-Pereira et al., 2023). In qualitative research examining interactions with an AI Virtual Mentor, students who provided richer contextual information and engaged iteratively described more positive experiences and more useful outputs than users who adopted a guarded, minimal-input approach (Syharat and Zaghi, 2026). These findings suggest that interaction quality plays a central role in shaping users' perceptions of usefulness in probabilistic systems.

These strands of research highlight a common pattern in which the quality of system outputs depends on the structure and completeness of user input. While these studies do not directly test the feedback mechanism proposed in this paper, they provide supporting evidence that interaction conditions can influence observed performance. This body of work motivates the need for a framework that explicitly accounts for how interaction behavior shapes performance and subsequent evaluation.

## 3 The guarded engagement loop

To move from reframing adoption to explaining its dynamics, the following section outlines the micro-level mechanism through which elevated risk salience reshapes trust and interaction in LLM use.

### 3.1 Trust and vulnerability

In organizational research, trust has been defined as a willingness to be vulnerable to another party's actions, based on the expectation that the other will perform an action that is important to the trustor, even in the absence of control over the other's actions (Mayer et al., 1995). Mayer et al. further argue that trust becomes meaningful in situations characterized by risk and uncertainty and is shaped by perceptions of ability, integrity, and benevolence. In automation contexts, trust shapes reliance decisions. Users must calibrate their level of trust to the system's actual capabilities because miscalibration can lead either to underuse or to misuse (Lee and See, 2004).

Users' feelings of vulnerability depend in part on their beliefs and feelings about AI. Emerging research suggests that user mindsets shape how they approach AI engagement. For example, studies of digital mindset and AI sensemaking indicate that openness to experimentation predicts educators' integration of AI tools, with trust serving as a baseline condition for adoption (Jiang and Meng, 2025). Similarly, research applying UTAUT to ChatGPT use finds that performance expectancy, effort expectancy, and social influence impact students' intentions to engage (Strzelecki and ElArabawy, 2024). Other work links mindset to AI-related anxiety and willingness to experiment, suggesting that beliefs and emotional responses influence how individuals approach AI systems (Dang and Liu, 2022; Kaya and Çelebi, 2025). These findings reinforce the idea that trust and perceived vulnerability, and thus the willingness to engage openly with AI systems, are mediated by users' beliefs, emotions, and prior experiences.

Producing high-quality outputs often requires users to disclose goals, constraints, partial reasoning, and sometimes sensitive contextual information. This creates exposure not only to the possibility of incorrect outputs, but also to concerns about how disclosed information may be stored, reused, or interpreted. In organizational settings where AI use is subject to scrutiny or debate, concerns about professional evaluation or accountability may further heighten this sense of vulnerability. When these perceived risks are high, users may limit contextual disclosure and iterative engagement. As discussed earlier, such constrained interaction can reduce output quality. Over time, diminished performance may erode trust, reinforcing more guarded use and limiting appropriate reliance (Lee and See, 2004). More broadly, patterns of reliance and trust calibration may vary depending on user experience and system design (Bansal et al., 2021; Buçinca et al., 2021).

### 3.2 Algorithm aversion

Empirical studies suggest that people can become algorithm averse. After observing an algorithm make mistakes, they may reject it even when it outperforms human judgment. Notably, equivalent errors can diminish confidence more for algorithms than for people (Dietvorst et al., 2015). However, subsequent work suggests that responses to algorithmic error are more variable and context-dependent than these early findings imply (Logg et al., 2019). Factors such as task type, the verifiability of outputs, user expertise, prior experience with AI systems, and how outputs are labeled or presented may all shape how errors are interpreted and whether they lead to rejection or continued use (Castelo et al., 2019; Logg et al., 2019; Zhang and Gosline, 2023).

In the context of LLM use, guarded engagement may increase the likelihood of low-quality outputs and visible errors and may therefore activate the dynamics of algorithm aversion. However, in contrast to traditional formulations of algorithm aversion, which often assume a shift from use to disuse following error exposure, interaction with LLMs allows for more nuanced behavioral responses. Rather than abandoning the system entirely, users may remain engaged while adjusting how they interact with it. Under such conditions, users who have not experienced the system under richer interaction conditions may be more likely to interpret these errors as evidence of system unreliability and may be less inclined to reengage or experiment further with generative AI. At the same time, the interactive nature of LLMs distinguishes them from the forecasting systems that dominate the algorithm aversion literature. Because users can revise prompts, steer outputs, and recover from errors, visible mistakes do not necessarily lead to rejection and may instead support learning, error recovery, and increasing user skill, depending on how users engage with the system. Users' reactions to such errors depend not only on their frequency but on how they are interpreted. In this framework, error exposure may not directly lead to rejection, but instead may shape users' interpretation of risks, which in turn may influence subsequent interaction behavior. In conversational AI contexts, visible mistakes may be attributed to randomness, superficial pattern matching, or a lack of underlying “understanding,” particularly when users lack a clear mental model of how probabilistic generation works (Kulesza et al., 2013; Yang et al., 2020). Because LLM outputs are often fluent and linguistically confident, errors may violate expectations of coherence or competence in especially salient ways. Such attributions can amplify perceptions that the system is unreliable, reinforcing dismissal rather than calibrated reassessment. It is worth noting, however, that most algorithm aversion research focused on forecasting systems rather than conversational models like LLMs. The extent to which the concept of algorithm aversion transfers to LLM contexts therefore remains an open question in need of further empirical research.

Such experiences may prompt users to compensate with overly cautious behaviors, including reduced experimentation or heightened monitoring, which further limit opportunities for trust calibration. Whether these responses result in continued but constrained use or in full disengagement is likely to depend on contextual factors such as how essential the system is, what alternatives are available, and whether users believe further interaction can improve outcomes. In terms of trust-in-automation, this pattern may reflect chronic under-reliance. Lee and See (2004) describe such patterns as disuse, a failure to rely on automation even when it may be appropriate. The guarded engagement loop can therefore be understood as one possible mechanism through which perceived risk, combined with early error exposure under constrained interaction conditions, shape not only whether users rely on generative AI, but how they engage with it over time. In this sense, the framework extends algorithm aversion by conceptualizing it not solely as disuse, but as a dynamic process of behavioral adaptation in which users may remain engaged while systematically constraining interaction.

### 3.3 Psychological safety and exploration

Research on psychological safety in organizations shows that people learn and contribute when they feel safe to take interpersonal risks; however, when the perceived cost of being wrong is high, they are less willing to explore or speak up (Edmondson, 1999). Even when interacting with a machine, concerns about privacy, accountability, or reputational consequences may make the perceived cost of experimentation more salient, resulting in a less iterative interaction style that hinders the development of competence.

### 3.4 The mechanism

The guarded engagement loop can be summarized as a reinforcing process. Perceived risk and low trust lead users to minimize disclosure and avoid iteration. Reduced interaction richness, in turn, lowers the usefulness of output and increases the likelihood of visible errors. The impact of these errors depends in part on how they are interpreted. When users lack a clear mental model of probabilistic, prompt-sensitive generation, visible mistakes may be attributed to incompetence or randomness rather than to constrained interaction conditions.

Observed errors may activate algorithm aversion and further erode trust. As trust declines, guarded behavior may increase, closing the loop. The process is self-confirming because protective interaction strategies alter user input in ways that produce the diminished performance users anticipated (Dietvorst et al., 2015). Mental model quality may therefore moderate the loop by shaping how performance signals are evaluated and how trust is recalibrated over time.

## 4 Safety and privacy as structural risks

Safety and privacy concerns should be treated as structurally relevant features of the adoption environment rather than as simple resistance. In many contexts, caution toward LLM use is rational given legitimate confidentiality and governance risks (NIST, 2023). The question becomes how this rational guardedness shapes interaction in ways that affect performance evaluations and trust calibration.

### 4.1 Privacy and disclosure

Privacy risks related to LLMs are not hypothetical. Research related to data extraction in LLMs shows that, when subject to targeted prompting, models can reproduce memorized verbatim text sequences, including strings of sensitive information (Carlini et al., 2021). Even when users are not adversarial, knowledge of memorization and extraction vulnerabilities can elevate perceived exposure risk, particularly when proprietary information is involved. This dynamic aligns with the privacy calculus framework, in which individuals weigh anticipated benefits against perceived privacy risks and signals of trustworthiness. When perceived risk is elevated or procedural fairness is uncertain, disclosure tends to decrease (Culnan and Armstrong, 1999). For LLMs, disclosure is directly tied to output quality, as contextual detail substantially influences performance. Privacy calculus therefore shapes both adoption decisions and the level of performance observed after adoption.

### 4.2 Security and safety threat models

Security guidance for LLM applications identifies several vulnerabilities that threaten security and safety, including prompt injection, insecure output handling, training data poisoning, and sensitive information disclosure (OWASP, 2023). These represent identifiable failure modes with practical implications, particularly when LLMs are embedded within organizational workflows, software systems, or decision processes.

Risk management frameworks increasingly treat these safety concerns as governance and engineering requirements rather than as optional best practices. The National Institute of Standards and Technology AI Risk Management Framework positions trustworthy AI as something built through ongoing risk identification, measurement, and mitigation, and it includes companion resources that profile risks and controls for generative AI systems (NIST, 2023). Likewise, ISO/IEC 42001 offers an AI management system standard intended to structure organizational governance around AI risks (ISO, 2023). This framework is operationalized through control clauses, lifecycle oversight, impact assessments, and documentation requirements, positioning AI-related risk as an ongoing organizational responsibility (Birogul et al., 2025). As these risks are codified within standards and management systems, potential harms are marked as institutionally salient features of the adoption environment.

### 4.3 Bounded openness

Because privacy and safety risks associated with generative AI are real and widely discussed, users may adopt protective strategies that limit disclosure and experimentation. The relevant question, then, is not simply how to encourage users to increase their openness and disclosure but rather how to structure disclosure within safe limits. In other words, adoption depends on enabling users to engage with AI with “bounded openness.” A central distinction is between a) genuinely high-risk disclosure (e.g., sensitive personal data, trade secrets) and b) low-risk disclosure critical for quality output (e.g., task goals, constraints, examples, or redacted contextual information).

The guarded engagement loop is most evident when high-stakes caution extends into lower-stakes contexts, limiting disclosure even in situations where risk is relatively low. This suggests that effective adoption depends on enabling users to engage in appropriately calibrated, policy-aligned disclosure, a capability shaped in part by organizational governance, technical safeguards, and institutional risk management processes consistent with contemporary AI governance frameworks (NIST, 2023; UNESCO, 2025).

### 4.4 Ethical and environmental risk

For some users, the most salient risk associated with generative AI is not data leakage or system failure, but ethical and environmental harms. Concerns about energy consumption, water use, supply chains, labor conditions, and the amplification of unjust systems contribute to a broader perception that AI use is embedded within infrastructures with ethically troubling social and environmental impacts. These concerns draw on analyses of environmental and social impacts associated with large-scale AI systems (Bender et al., 2021; Luccioni et al., 2023), even as some scholars note the potential for AI applications to contribute to energy optimization and climate mitigation (Patterson et al., 2021; Rolnick et al., 2019).

When ethical and environmental risk becomes central, the dynamic resembles the guarded engagement pattern described above. Users who perceive AI use as ethically problematic may limit engagement, avoid experimentation, or disengage entirely. This stance can shape not only whether the system is used, but how it is used. Avoidant users may engage in ways characterized by minimal iteration, limited integration, and reluctance to explore higher-leverage applications. This produces interaction conditions similar to those associated with privacy concerns, resulting in lower-quality outcomes that may reinforce skepticism about the system's legitimacy or value. In terms of trust-in-automation, this represents disuse, defined as under-reliance on a system capable of providing value under appropriate conditions (Lee and See, 2004).

Alternately, some ethically motivated users may withdraw from AI use altogether. While Lee and See (2004) describe disuse primarily at the level of individual engagement, values-driven withdrawal can also operate at a collective level. Public discourse about AI reflects the uses that become visible through active participation. If ethically oriented individuals choose not to engage with AI, justice-oriented or public-interest applications may be underrepresented in that visible landscape. Commercial or ethically ambiguous uses may then become more prominent, reinforcing the perception that AI primarily serves those purposes. In contrast, diverse examples of successful AI use may recalibrate perceived risk by demonstrating that AI can generate value in ways consistent with institutional norms and ethical priorities. Such examples may temper overgeneralized guardedness without dismissing legitimate concerns.

### 4.5 Technological uncertainty

Uncertainty about the direction and pace of AI development may also function as a source of perceived risk. As users recognize rapid changes in model capability, regulation and oversight, and even social norms, they may respond with caution, limiting engagement as they attempt to understand the implications of engagement with the technology.

## 5 Social amplification of risk

Safety and privacy concerns do not form in a vacuum. Risk perception is socially constructed through institutions, culture, and communication environments. The social amplification of risk framework (SARF) describes the news media and related networks as “amplification stations” that transmit, interpret, and magnify risk signals, sometimes shaping public response beyond technical risk estimates (Kasperson et al., 1988).

### 5.1 Media framing in AI coverage

Empirical analyses of AI news content provide a nuanced picture. A large-scale study of global media reporting found that while most AI-related headlines were neutral in sentiment, negative headlines outnumbered positive ones among those that were not (Ittefaq et al., 2025). Regional differences were also evident in the study's findings, with Global North outlets framing AI negatively more often (about 24%) and emphasizing innovation less, while Global South outlets placed greater emphasis on innovation and less on ethical and privacy concerns. Similarly, a 2025 report shows that only 39% of people in the United States hold optimistic views about AI, as compared to 83% of those in China (Stanford HAI, 2025). Separately, a content analysis of AI coverage in major United States and United Kingdom outlets found that reporting frequently links AI to distinct data risks including bias, surveillance, cyber-conflict, and information disorder, suggesting that the salience of potential harms may be shaped by framing patterns even when overall sentiment appears neutral (Nguyen and Hekman, 2024).

At the same time, claims of uniformly negative media bias over long spans of time are not supported. A longitudinal analysis of 30 years of New York Times coverage found that AI reporting was consistently more optimistic than pessimistic overall, even as content related to existential and ethical concerns (i.e., loss of control, ethical use, and job displacement) increased in prominence over time (Fast and Horvitz, 2017). While the study considered literature just prior to the AI boom of the early 2020s, the data points to the increasing prevalence of risk narratives within headlines over time. The implication is therefore not that media tone is uniformly negative, but that distinct risk narratives can grow in salience even within a broader discourse that includes neutral and optimistic narratives.

### 5.2 Negativity bias and risk salience

Two well-established dynamics suggest that even modest levels of negativity can shape perception disproportionately. First, research on negativity bias demonstrates that negative information tends to have stronger effects on cognition and behavior than positive information (Baumeister et al., 2001). Second, evidence indicates that negatively worded headlines increase engagement and consumption, creating financial incentives for risk- and conflict-oriented framing in competitive media environments (Robertson et al., 2023). These dynamics suggest that even in a media environment characterized by neutral average tone, risk-related cues may become disproportionately salient, contributing to a public understanding of AI organized around concerns about safety, surveillance, and misinformation (Kasperson et al., 1988).

### 5.3 Public concern about AI

Recent survey work indicates that, in the United States, many people are more concerned than excited about the increased use of AI, and most want more control over how it is used in their lives (Pew Research Center, 2025). A Bentley University-Gallup study found that more Americans perceive AI as harmful than beneficial and that majorities express low trust in businesses to use it responsibly; respondents also indicated that transparency about AI use is the most effective way for organizations to address their concerns (Gallup, 2024). Additionally, the Stanford AI Index reports that confidence in AI companies' ability to protect personal data declined from 50% in 2023 to 47% in 2024, reflecting a broad erosion of privacy trust across regions (Stanford HAI, 2025).

These public attitudes matter because they shape interactions with AI. When individuals are primed, whether through risk-oriented framing, public discourse, or institutional norms, to perceive LLMs as privacy risks, they may choose to limit disclosure. Because contextual disclosure is central to output quality, this protective strategy can lower performance and increase the likelihood of unsatisfactory outcomes. These experiences may then reinforce distrust. Risk salience therefore affects not only adoption rates but also the quality of outcomes among adopters, with the potential to reinforce negative beliefs through use (Culnan and Armstrong, 1999).

### 5.4 Legitimacy, stigma, and asymmetric risk framing

Adoption dynamics are also shaped by institutional legitimacy, the generalized perception that an activity is appropriate and aligned with prevailing norms and values within a social system (Suchman, 1995). When the legitimacy of AI use is contested, adoption is evaluated not only in terms of tool performance, but also in terms of reputational, moral, and professional risk (Lee and See, 2004).

This dynamic is seen in domains such as higher education, where generative AI has been framed simultaneously as a productivity tool and as a threat to academic integrity and learning (Kasneci et al., 2023). Institutional responses have ranged from experimentation to restriction, indicating that the boundaries of acceptable use still lack consensus. In such environments, AI stigma can emerge. Drawing on stigma theory (Goffman, 1963), stigma refers to a socially constructed mark that renders an activity or attribute discrediting in a given context. AI stigma, in this sense, refers to the perception that using AI is ethically questionable, intellectually lazy, environmentally irresponsible, or professionally unacceptable. Recent work describes “AI shaming” in academic contexts, in which researchers are criticized or dismissed for using AI tools, reinforcing the perception that AI-assisted work is less authentic or legitimate (Giray, 2024). Because stigma operates through anticipated judgment, individuals may moderate their behavior not because use is prohibited, but because it is perceived as socially or professionally risky.

The anticipation of social risk then creates an asymmetry between use and disuse. The risks associated with using AI, such as privacy breaches, bias, misinformation, or professional consequences, are concrete and visible. By contrast, the risks of not using AI, such as lost efficiency or missed innovation, are less visible and more hypothetical. Prospect theory suggests that potential losses tend to carry more weight than equivalent gains (Kahneman and Tversky, 1979). When AI use is understood as exposure to reputational or moral loss, and non-use is framed as foregoing potential benefits, the perceived downside of use can dominate decision-making even when trade-offs are unclear.

While AI stigma may shape engagement in the current context, its persistence over time remains uncertain. As generative AI systems improve and become more widely adopted, the perceived costs of use may decline and, in some cases, may even reverse. At the same time, the consequences of not using AI, such as reduced efficiency or competitive disadvantage, may become more salient. Together, these shifts suggest that the current imbalance between the perceived risks of use and non-use may diminish over time. This asymmetry helps explain patterns of ethical withdrawal. When AI use is perceived as discrediting or misaligned with institutional or social norms, individuals may limit experimentation or disengage altogether. Such withdrawal may be principled, but it reduces opportunities for experiential learning and makes trust harder to recalibrate. In this way, legitimacy contestation and AI stigma increase the salience of potential harms before any direct interaction takes place (Kasperson et al., 1988). Legitimacy concerns may therefore contribute not only to ethical withdrawal, but also to more guarded forms of engagement among those who continue to use the system. This extension is intended as a speculative component of the framework rather than an empirically established mechanism. It highlights a possible pathway through which patterns of use and non-use may shape engagement and learning dynamics, but this relationship remains an open question for future research.

These dynamics are particularly visible in research and scholarly contexts, where the use of generative AI tools is often subject to scrutiny and evolving norms. AI-assisted writing, analysis, and peer review raise questions about authorship, originality, and evaluation, and may be judged differently depending on how such tools are disclosed and perceived. As a result, legitimacy, trust, and stigma play a direct role in shaping how AI-supported research outputs are evaluated within academic and professional communities.

## 6 A multilevel model of guarded engagement

**Figure 1:**
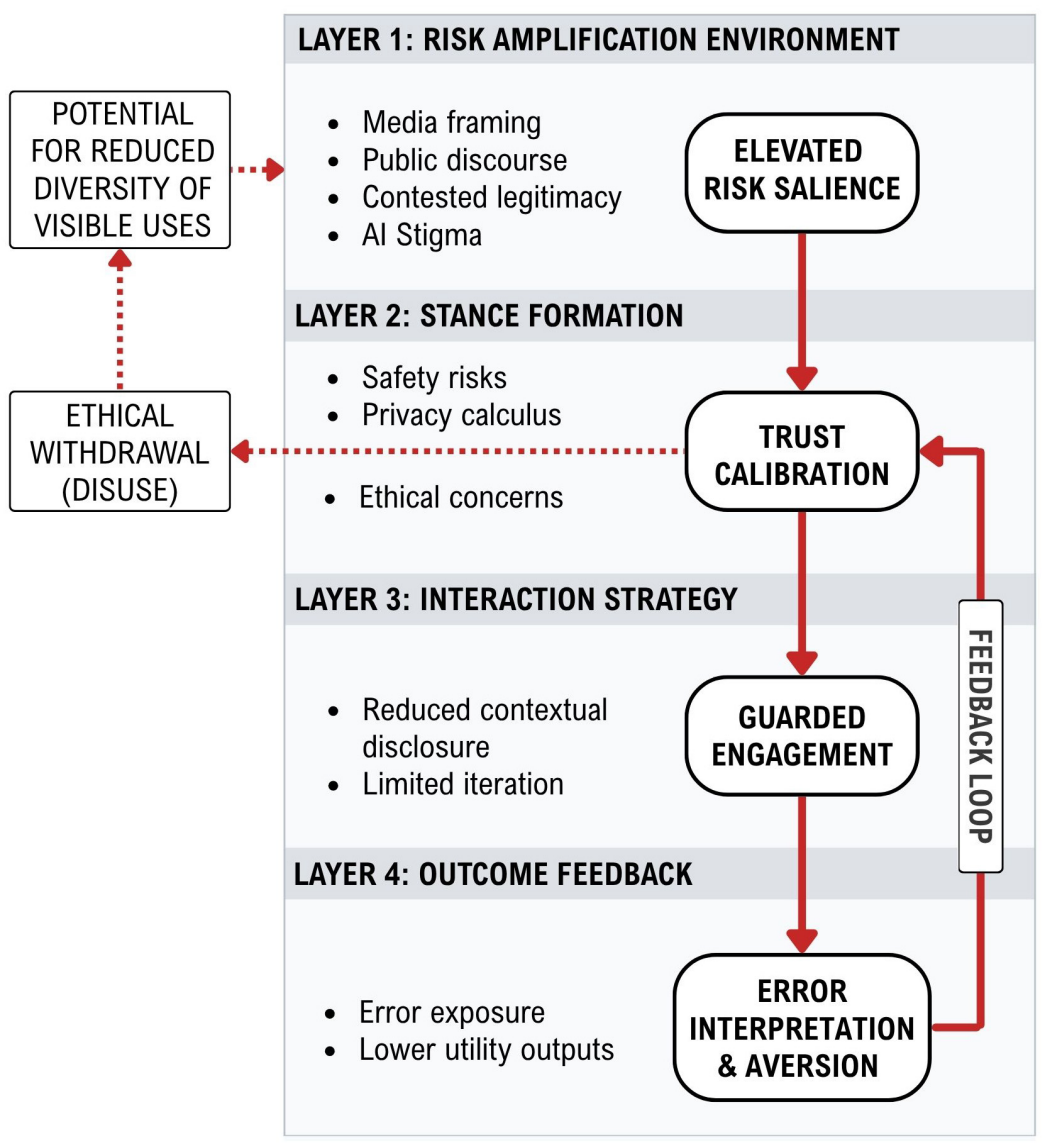
The Guarded Engagement Loop: A conceptual framework of risk-driven AI underperformance. The framework proposes that elevated risk salience shapes trust calibration and encourages guarded engagement strategies, such as reduced contextual disclosure and limited iteration. These interaction constraints may increase error exposure and reduce output utility, reinforcing distrust through a feedback loop. Dashed arrows indicate potential pathways to ethical withdrawal (disuse) and reduced diversity of visible AI use cases.

Risk salience in LLM adoption does not originate solely from direct interaction with the system. It is shaped by multiple, interacting environments. At the micro level, individual experiences with outputs and errors influence trust calibration. At the institutional level, legitimacy contestation and AI stigma shape the perceived social and moral risks of use. At the informational level, media framing and public discourse amplify certain risk narratives and render them more visible than countervailing benefits (Kasperson et al., 1988). These influences create an amplified risk environment within which user-system interaction unfolds. Within this environment, the effect of risk salience on engagement is shaped jointly by the type of risk perceived, the context of use, and the user's prior experience, which together influence whether users disengage, adopt guarded engagement, or engage more openly. Both the guarded engagement loop described earlier, and patterns of ethical withdrawal operate inside this broader field of socially structured risk perception. The model proposes a reinforcing dynamic in which amplified risk salience is expected to shape user stance toward AI, influencing interaction strategy which in turn shapes the conditions under which performance is produced and evaluated, and subsequently informs trust calibration. Figure 1 visualizes this multilevel feedback structure.

The feedback loop described here is intended as a proposed mechanism, and its strength and persistence are likely to vary across users, tasks, and contexts. The framework specifies four interrelated layers through which risk perceptions translate into engagement patterns and feedback effects:

**Layer 1–Risk amplification environment:** Media framing, institutional controversy, public discourse, and legitimacy contestation shape a risk-amplifying environment that elevates the salience of AI-related harms prior to interaction (Kasperson et al., 1988).

**Layer 2–Stance formation:** Users interpret the AI system through the lens of trust-in-automation and privacy calculus, while also incorporating perceived safety risks and ethical concerns, forming a stance on whether and how much to rely on the system (Lee and See, 2004). In contexts where legitimacy is contested, this stance may result either in guarded engagement or in ethical withdrawal prior to interaction.

**Layer 3–Interaction strategy and conditions:** For those who engage, user stance shapes interaction behavior, including the degree of disclosure and iteration. These behaviors, in turn, determine interaction richness, understood as the informational and iterative depth of the user-AI exchange. Prompting scholarship and human-AI interaction guidelines indicate that iterative and structured engagement can materially influence model performance (Wei et al., 2022). Guarded interaction reduces contextual disclosure and limits iterative refinement, resulting in lower interaction richness.

**Layer 4–Performance and outcome feedback**: Because AI output quality depends on the conditions of interaction, interaction richness shapes both objective performance and perceived performance. Lower interaction richness is associated with lower-quality outputs and a greater likelihood of visible errors. These outcomes are interpreted by users and feed back into trust calibration through aversion-like dynamics, although the strength and form of this mechanism may vary in interactive LLM contexts (Dietvorst et al., 2015). Reduced performance under constrained interaction conditions may reinforce guarded engagement, while high-quality outcomes may recalibrate trust and reduce perceived risk.

Ethical withdrawal and guarded engagement therefore represent two related but distinct responses to amplified risk salience. Withdrawal reduces opportunities for experiential learning and limits recalibration of trust through direct feedback. In contrast, guarded engagement maintains interaction with constraints, shaping the interaction in ways that may reduce performance and reinforce distrust. Both pathways are embedded within the same amplified risk environment.

The strength of the guarded engagement loop is likely to vary across contexts and users. The dynamics described here are expected to be most pronounced in settings where perceived risks are high, interaction requires contextual disclosure, and users have limited prior experience or well-developed mental models of the system. By contrast, the loop may be weaker in contexts where tasks are highly verifiable, users have greater expertise, or organizational conditions support experimentation and learning. These conditions play distinct roles within the proposed model: risk type shapes what users are concerned about, task and institutional context shape how consequential potential errors or disclosures are, and prior experience shapes how controllable and interpretable the system is perceived to be. In addition, different types of risk may shape behavior in distinct ways. Privacy and security concerns may primarily constrain disclosure, while ethical or environmental concerns may lead to selective engagement or withdrawal. These factors are anticipated to determine not only the intensity of guarded engagement, but also the form it takes and its downstream effects on interaction richness and performance. For example, high perceived risk in combination with low prior experience may amplify guarded engagement, whereas similar risks under conditions of high experience and clear governance may instead support more iterative and exploratory use. These variations suggest that the guarded engagement loop should be understood as a context-dependent mechanism rather than a universal pattern of use.

### 6.1 Propositions

The guarded engagement loop should be understood not only as a model of individual adoption, but also as a framework with implications for research metrics and analytics. In contexts where generative AI is used in scholarly writing, analysis, peer review, and evaluation, interaction conditions may shape the performance evidence used to assess the quality, reliability, and legitimacy of AI-assisted outputs. The model above yields a set of propositions that can guide empirical studies and identification of adoption barriers in probabilistic systems.

**Proposition 1 (Legitimacy contestation increases ethical withdrawal**): Contested legitimacy or AI stigma increases the likelihood of ethical withdrawal prior to interaction (Giray, 2024; Goffman, 1963; Suchman, 1995).

**Proposition 2 (Perceived risk increases guarded engagement and reduces interaction richness):** Higher perceived privacy and safety risk increases the likelihood that users adopt guarded engagement strategies, which in turn reduce interaction richness by limiting contextual disclosure and iterative refinement (Culnan and Armstrong, 1999).

**Proposition 3 (Interaction richness shapes performance outcomes):** Lower interaction richness is associated with lower objective performance and lower perceived performance, as the quality of AI outputs depends on the structure, context, and iteration present in the interaction (Liu et al., 2023).

**Proposition 4 (Error exposure under constrained interaction increases algorithm aversion):** Exposure to errors under constrained interaction conditions is associated with reduced reliance on generative AI systems, particularly when users interpret these errors as evidence of unreliability. This relationship is likely to vary depending on task type, output verifiability, user expertise, prior experience, and system presentation. In interactive contexts, error exposure may also support learning and trust calibration rather than rejection (Castelo et al., 2019; Dietvorst et al., 2015; Logg et al., 2019).

**Proposition 5 (Risk-focused media framing amplifies perceived risk):** Greater exposure to AI-related media framing that emphasizes surveillance, privacy, cybersecurity, or misinformation risks increases perceived privacy and safety risk, reinforcing guarded engagement and reduced interaction richness (Nguyen and Hekman, 2024).

**Proposition 6 (Governance clarity enables bounded openness and improves interaction conditions):** Clear and credible governance, including policies, safeguards, and risk management frameworks, reduces overgeneralized guardedness and enables forms of bounded openness that support higher interaction richness without increasing unsafe disclosure (NIST, 2023).

These propositions clarify and sharpen the core claim that prompting difficulty alone does not account for adoption barriers. Rather, the barrier operates as a multilevel system in which risk perceptions shape interaction behavior, interaction behavior shapes experienced performance, and experienced performance reshapes belief.

The propositions outlined here lend themselves to empirical testing using a range of study designs. For example, experimental studies could manipulate risk salience and disclosure constraints to examine their effects on interaction richness, output quality, and trust calibration. Observational or field studies could analyze patterns of iteration, contextual disclosure, and reuse behavior in real-world LLM use. Such approaches would allow researchers to examine how interaction conditions shape performance and evaluation across different users, tasks, and settings.

## 7 Institutional and design implications

The framework is particularly relevant to research metrics and analytics, where generative AI is increasingly used in scholarly writing, coding, literature synthesis, peer review, and research evaluation. In these contexts, guarded engagement may appear as reluctance to share project-specific context with AI tools, avoidance of AI assistance even for permitted low-risk tasks, or concealment of AI use due to stigma or unclear disclosure norms. These behaviors can shape not only individual productivity but also the performance evidence used to evaluate whether AI improves scholarly quality, peer-review reliability, and research workflows. As a result, legitimacy, trust, and stigma do not operate only at the level of adoption, but also in the evaluation of AI-assisted research outputs, where disclosure of AI use may influence perceptions of quality, credibility, and acceptability. The guarded engagement loop therefore provides a lens for understanding how interaction conditions, institutional norms, and evaluation practices co-produce the evidence used to assess the role of AI in research.

### 7.1 Institutional safeguards

If guarded engagement is partly driven by rational privacy and security concerns, then changes in user behavior depend on changes in institutional conditions rather than on informal assurances from AI providers, administrators, or policymakers. Risk management frameworks offer a structured approach. The NIST AI Risk Management Framework and its generative AI profile emphasize governance across the AI lifecycle, including risk identification, measurement, mitigation, auditing, and accountability (NIST, 2023).

In practice, this involves establishing environments in which experimentation is formally supported. Formally approved testing environments, clearly defined use cases, and explicit guidance on anonymizing sensitive information or using synthetic examples allow users to provide enough context to learn effective interaction strategies without undue disclosure risk. Such arrangements make bounded openness structurally possible. This logic parallels research on psychological safety, which shows that learning improves when individuals can experiment without disproportionate reputational or professional penalty (Edmondson, 1999).

### 7.2 Designing for bounded openness

Design guidance for human-AI interaction emphasizes that systems should help users understand system capabilities, identify and correct errors, and recover from failures, thereby lowering the perceived stakes of experimentation (Amershi et al., 2019). For LLM systems, privacy-aware defaults may include contextual prompts before sensitive entries, structured inputs that encourage non-sensitive constraints first, and features that support progressive disclosure through anonymized summaries before additional detail is added. From a security perspective, OWASP's LLM Top 10 identifies prompt injection and sensitive information disclosure as predictable risk classes. Incorporating validation, input controls, and access restrictions into LLM applications can reduce actual incidents and, in turn, reduce perceived risk, thereby loosening the guarded engagement loop (OWASP, 2023).

However, model capability may also shape the dynamics described here. Users who are more skeptical of AI may be less willing to access higher-capability systems, resulting in interactions with models that handle missing context less effectively. In such cases, differences in model capability may amplify the effects of constrained interaction or contribute to lower observed performance independently of interaction strategy. This suggests that access to model capability is an additional condition that may influence how bounded openness translates into effective use.

### 7.3 Risk communication

Because media environments amplify risk salience, organizations benefit from communication strategies that acknowledge and differentiate among types of risk. Effective communication clarifies what specific safety and privacy risks exist, what uses are prohibited, what uses are permitted, and what safeguards are in place. Such an approach aligns with established principles of risk communication and reduces the risk of backlash if users perceive that risks were downplayed. The objective is not unqualified trust, but calibrated reliance under transparent constraints (Lee and See, 2004).

## 8 Conclusion

Generative AI adoption is often framed primarily as a question of prompting skill. A more complete explanation considers how poor outcomes can emerge from feedback dynamics operating within environments where risks are highly salient. In some contexts, perceived misalignment of the technology with personal values, legitimacy contestation and AI stigma generate ethical withdrawal prior to engagement, limiting opportunities for trust recalibration through experience and experimentation and sustaining elevated perceptions of risk. For those who do engage, heightened risk salience, particularly around privacy and safety, may encourage guarded interaction. When engagement becomes guarded, interaction richness may decline, increasing the likelihood of low-utility outputs and visible errors. These experiences may then trigger algorithm aversion, further reducing trust and reliance. This loop may be reinforced by media and discourse environments that, even when not uniformly negative, foreground risk frames that make protective stances psychologically and institutionally rational. By specifying how risk perceptions may recursively shape performance through interaction strategy, the guarded engagement loop extends existing trust and adoption models to account for the distinctive properties of probabilistic, interaction-dependent AI systems.

The implication is not that skepticism is misplaced, but that governance structures and secure system design can translate legitimate caution into conditions that support bounded openness and psychologically safe experimentation. Under such conditions, individuals and institutions are better positioned to evaluate generative AI based on its actual capabilities rather than on the underperformance that guarded interaction can produce (Dietvorst et al., 2015). The propositions in this paper may be tested in future empirical research that examines how variations in risk perception, legitimacy contestation, organizational policy, and media exposure predict interaction richness and subsequent perceived usefulness and continued use. Intervention studies comparing bounded openness protocols against standard training could clarify the practical value of the framework.

The guarded engagement dynamic is not inevitable. It emerges from the interaction of technical design, institutional conditions, and communication environments. Recognizing these dynamics highlights how adoption outcomes are shaped not only by technological capability but also by the conditions that structure engagement. As generative AI becomes more integrated into education, healthcare, professional services, and public life, understanding these dynamics will become increasingly important.

## Data Availability

The original contributions presented in the study are included in the article/supplementary material, further inquiries can be directed to the corresponding authors.
